# Decreased blood pressure among community dwelling older adults following progressive muscle relaxation and music therapy (RESIK)

**DOI:** 10.1186/s12912-019-0357-8

**Published:** 2019-08-16

**Authors:** Niken Fitri Astuti, Etty Rekawati, Dwi Nurviyandari Kusuma Wati

**Affiliations:** Faculty of Nursing, Universitas Indonesia, Jalan. Prof.Dr.Bahder Djohan,Kampus UI, Depok, West Java 16424 Indonesia

**Keywords:** Blood pressure, Hypertension, Music therapy, Older adults, Progressive muscle relaxation

## Abstract

**Background:**

Hypertension is a major risk factor related to leading causes of death among older adults. Numerous efforts have been done but they still remain sub-optimal. This condition encourages development of a non-pharmacological therapy to complement pharmacological therapy, such as progressive muscle relaxation and music therapy (RESIK). The purpose of this study was to determine the effect of RESIK on blood pressure among older adults with hypertension in Depok Indonesia.

**Methods:**

This study used quasi experimental design with pre and post test with control group approach. One-hundred older adults with hypertension were divided into two groups using stratified random sampling and purposive sampling.

**Result:**

After 11 sessions of RESIK therapy in 6 days, the paired t-test showed a decrease in systolic blood pressure to 29.2 mmHg and a decrease in diastolic blood pressure to 16.2 mmHg.

**Conclusion:**

In conclusion, RESIK decreased systolic blood pressure *p* < 0,001, but it did not significantly decrease diastolic blood pressure *p* > 0.167. It is recommended that RESIK be used regularly for an older adult population with hypertension.

## Background

Hypertension is one of the leading causes of death, worldwide. As many as 9.4 million people die every year due to hypertension, and more than 1 billion people live with high blood pressure, 40% of which are over the age of 25 [1]. It was estimated 25.8% of Indonesia’s population at 2013 suffered from hypertension [2]. Worldwide, hypertension is a major factor in the cause of death among older adults due to conditions, such as ischemic heart disease and stroke [3]. Moreover, the high prevalence of this disease can affect a country’s economy [4].

The Indonesian government has undertaken efforts to surveillance of Non-Communicable Diseases (NCDs) based Posbindu and pharmacological therapy [5]. However, both efforts did not have optimal impact, a study explained the evaluation of surveillance of NCDs risk factors based on the Posbindu NCDs resulted the surveillance personnel, facilities, and financing were in accordance with the guidelines, data collection was conducted but the data processing, data analysis, data interpretation and dissemination of information was not conducted during surveillance, the coverage of examination at Posbindu and Puskesmas level was less than cut off point [6]. Other studies also explained that the treatment of hypertensive patients had not been effective because of recurrence andlong term side effects [7] . Example, the older adults consumed angiotensin-converting enzyme (ACE) at risk of new-onset osteoporotic fracture (NOF) [8]. These conditions encouraged the development of non-pharmacologic therapies to complement pharmacological therapeutics, thereby enhancing treatment outcomes [7].

Non-pharmacologic therapy, such as progressive muscle relaxation and music therapy (RESIK), is a type of nursing intervention used to decrease blood pressure [9]. Many studies have explained the effect of progressive muscle relaxation on blood pressure, but this type of therapy would be better if it is done simultaneously with other types of relaxation therapy, such as music therapy. In fact, breathing exercises, progressive muscle relaxation and music therapy have been found to decrease blood pressure, especially in older adults [10].

This research used music therapy in conjunction with progressive muscle relaxation. Music therapy was a therapy that could help in the healing process. Music that could be used in this therapy was a consistent and stable rhythm, dynamic, fun harmonious, regular rhythm without any sudden change [11]. The instrumental music of Peter Sterling’s “The Angels Gift” was one of the instrumental music of the harp, flute, violin and soft orchestral strings that could stabilize blood pressure after being given for 25 min [12]. In the current studies, there had been no study that combined progressive muscle relaxation and music therapy (RESIK). Therefore, the researcher was motivated to explore the effect of progressive muscle relaxation and music therapy (RESIK) to decreased blood pressure among older adults with hypertension. So this therapy could be one of alternatives that could be given to the older adults with hypertension.

## Method

### Study design and sample

**Fig. 1:**
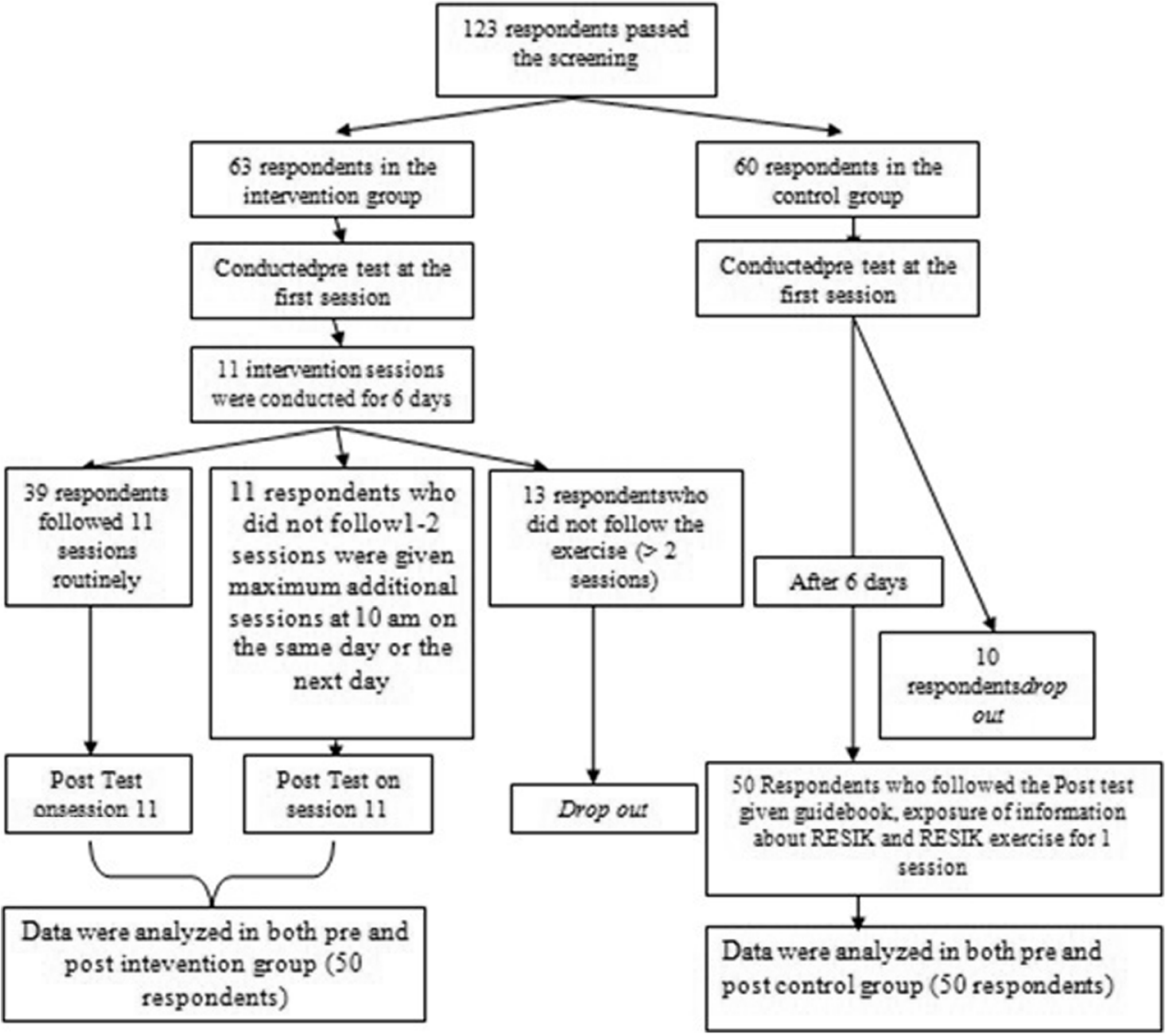
Sampling procedure

This study used quasi experimental design with pre and post test with control group approach. In the present study, the sample consisted of 100 older adults with hypertension in Depok, Indonesia. The participants were divided into an intervention group and a control group. The sample size in the study was determined using different formula 2 mean dependent group that had considered the drop out sample and the design effect. Sampling method used stratified random sampling. Researcher conducted two times drawing, the first drawing was to determine the research area, from 11 districts in Depok City, researcher got Cimanggis as a place for research, followed by the second drawing which aimed to determine the district used as the intervention group as well as the control group. Results from the second drawing, from 6 Sub-districts in Cimanggis, Harjamukti Sub-district was obtained as research area for intervention group and Cisalak Pasar for control group, next the researcher used purposive sampling to choose community groups which were used as research place. The inclusion criteria were: older adults with systolic blood pressure ≥ 140 mmHg and diastolic ≥90 mmHg, with no hearing and vision impairment, no mobilisation impairment or cognitive disorder, with a mini-mental state exam (MMSE) score ranging from 18 to 23 and who had or had not undergone standard anti-hypertension therapy. The exclusion criterion was older adults with critical hypertension, characteris ed. by systolic blood pressure ≥ 180 mmHg or diastolic blood pressure ≥ 120 mmHg.Sampling procedure can be seen in Fig. 1.

### Procedure

The study was conducted as many as 11 sessions in 6 days in the morning and evening with 3 practice sessions. The exercise schedule took one week to get maximum result [13–15]. Six day interventions with a duration of 15 min significantly reduced systolic blood pressure in primary hypertension [16]. The data analysis used paired t-test and independent t-test.

The present study has received ethical clearance and was approved by the Faculty of Nursing Ethical Committee of the Universitas Indonesia on 17 April 2017. The intervention caused no harmful side effects for the respondent if the respondent did in accordance with the procedure, but if the respondent did not follow the procedure accordingly, blood pressure would increase. If the respondents’ blood pressure increased during therapy, they were encouraged to rest while breathing regularly for 5 min until they felt relaxed.

## Result

The mean age of the respondents in the intervention group was 66.54 (standard deviation [SD] = 5.665). In the control group, the mean age of the respondents was 67.48 (SD = 5.388) with a homogeneity score of 0.397. All the characteristic variables were tested for homogeneity using a chi-square test for the categorical data and an independent t-test for the numerical data. The results showed that the respondents characteristics in the intervention and control groups were homogeneous. Other respondent characteristics are presented in Table 1.

**Table 1:** Respondent characteristic

Characteristic	Intervention (*n* = 50)	Control (*n* = 50)	*P*
	f (%)	f (%)	
Gender, Female (%)	30 (60)	32 (64)	0.680
Education level, Senior high school (%)	27 (54)	17 (34)	0.252
BMI, Normal (%)	26 (52)	24 (48)	0.100
Family History, No (%)	26 (52)	35 (70)	0.065
Smoking History, No (%)	41 (82)	40 (80)	0.799
Anti-Hypertensive Medication,Yes (%)	34 (68)	37 (74)	0.509

Note: The characteristic of respondents were the highest precentage. All variables were homogeneous and had been tested with chi square test. *BMI* Body Mass Index

## Discussion

Participating in progresive muscle relaxation until feeling relaxed, calm and while fully concentrating for 30 min can decrease a person’s corticotrophin releasing hormone (CRH) and adrenocorticotropic hormone (ACTH) levels in the hypothalamus [17]. This process decreases sympathetic nerve activity, so adrenaline and non-adrenaline levels can also decrease. This resulted in a decreased heart rate, widening of the blood vessels, decreased blood vessel resistance and decreased exertion of cardiac muscles, thus, decreasing cardiac arterial blood pressure [17].

This study showed that blood pressure decreased as many as 29.2 mmHg (systolic) and 16.2 mmHg (diastolic), the detailed results are scribed in Table 2. Another research showed a progressive muscle relaxation which have been done by 40 respondents aged 40–70 years for 8 sessions in 4 days with duration 20 min in the morning and afternoon could only decrease blood pressure as many as 6.42 mmHg (systolic) and 0.8 mmHg (diastolic) [18]. That condition was due to the combination between progressive muscle relaxation and music therapy.

**Table 2:** Differences of blood pressure before and after intervention in the intervention and control group

Variable	Mean		SD**		Mean different	*P*
	Pre	Post	Pre	Post		
Systolic						0.001*
•Intervention	152.8	123.6	11.61	14.81	29.2	
•Control	156.2	142.2	11.76	15.82	14.0	
Diastolic						0.001*
•Intervention	96.40	80.20	7.217	11.16	16.2	
•Control	96.60	83.20	7.453	10.39	13.4	

*statistically significant on on α < 0.05**deviation standard

Showed the changes of blood pressure before and after intervention in both group (*p* < 0.05), with the highest change in the intervention group which systolic was 29.2 mmHg and diastolic was 16.2 mmHg

The research suggested that music could inhibit and balance brain waves, capable to activate limbic system related with emotion. When the limbic system was activated, the individual would feel relaxed. The music could affect sympathoadrenergic activities that had a role in plasma catecholamine concentrations and also affected the release of stress-released hormones and stimulated the body to produce nitric oxide (NO) molecules that working on blood vessel tone and could decrease blood pressure [19–21].

Changes in blood pressure are related to the administration of anti-hypertensive drugs, which are routinely used by older adults. Research has shown that captopril can decrease systolic blood pressure and diastolic blood pressure by as much as 29.16/11.83 mmHg and amlodipine can decrease systolic blood pressure and diastolic blood pressure by as much as 32.94/16.38 mmHg [22]. Based on those findings, the decrease in blood pressure in the present study was caused by both the RESIK therapy and the anti-hypertensive drugs taken by the respondents. If the effect of taking anti-hypertension drugs for most of the respondents (68%) had a minimum effect on blood pressure (as much as 29.16/11.83 mmHg compared with the blood pressure final result, which was as much as 29.2/16.2 mmHg), it could be concluded that RESIK therapy could be combined with pharmacological therapy to help ensure a maximum decrease in blood pressure.

(Table 3) shows none significant differencein diastolic blood pressure after RESIK therapy for 11 sessions. Diastolic hypertension is often associated with a decrease in cardiac muscle function, the ability of the heart to pump and stiffness of the heart muscle [16]. A person’s age also physiologically affects cardiac function. When cardiovascular function decreases, the ability of the heart to pump and stiffness in the heart muscle causes diastolic blood pressure to decrease, but not significantly. In contrast, in systolic hypertension cardiovascular function can progressively change due to changes in the elasticity of the blood vessels, so systolic blood pressure can change more rapidly than diastolic blood pressure.

**Table 3:** The differences of blood pressure before and after intervention in the intervention and control group

Variable	Mean	SD**	*P*
Systolic			
•Intervention	123.6	14.81	0.001*
•Control	142.2	15.82	
Diastolic			
•Intervention	80.20	11.16	0.167
•Control	83.20	10.39	

*meaningful on α < 0.05 ** deviation standard

Illustrated that there was a significant difference of systolic blood pressure between intervention and control group (*p* < 0.05), but there was no differences of diastolic blood pressure between intervention and control group (*p* > 0.05)

Other factors that may affect diastolic blood pressure include tea drinking habits, triglyceride levels, lipoproteins, blood glucose and Body Mass Index (BMI) [23]. This finding is in line with the results of this study, which found that 29% of the respondents had a BMI with overweight categories. Another study reported that direct and indirect exposure to cigarettes could increase a person’s heart rate by as much as 34% and diastolic blood pressure by as much as 17% [24]. This study was in line with the results of this study, that blood pressure did not decrease in 5 of the 9 respondents in the intervention group who had a smoking history, so it could be concluded that many factors could affect diastolic blood pressure. The above conditions could be minimised by modifying a person’s lifestyle (lifestyle modification in JNC VII), such as losing weight, adopting a healthy diet, restricting daily salt intake, increasing physical activity, restricting alcohol consumption and stopping smoking [25].

In the present study, blood pressure was sometimes increased after treatment. This outcome occurred in 5 respondents because 4 of them did not follow the protocol, such as exhaling through the mouth, and they remained tense when the researchers instructed them to relax their muscles and 1 respondent felt worried about her blood pressure. The intervention protocol indicates that while respondents are attempting to relax, nurses or researchers must consider how to relax the muscle group a respondent is tensing. If respondents do not achieve a relaxed state, CRH is secreted and ACTH levels in the hypothalamus are not optimal, so the parasympathetic nervous system releases the neurotransmitter acetylcholine to inhibit the sympathetic nerves by reducing the contractile heart muscle; moreover, vasodilation of the anterior and vein was also not optimal so the blood pressure could not significantly decrease [26].

RESIK therapy has to be done regularly to maintain blood pressure within normal limits. RESIK in this study was a coping mechanism regulator that could affect physiological function, self concept, role function and interdependensi elderly. These effects would provide both adaptive and ineffective responses [16]. Roy’s theory explained that an individual could improve his health by maintaining behavior adaptively and being able to change behavior maladaptif. The constant interaction between humans and the environment would have an impact on internal and external changes, so did treatment of adaptive behavior continously in the elderly with high blood pressure caould be formed [27].

Evidently, the mean of systolic and diastolic blood pressure in the intervention group included in the pre-hypertension category. That was different with control group. The mean of blood pressure in the control group was in category 1 hypertension. While diastolic blood pressure was no difference significantly with the control group, this therapy could help maximizing the treatment that has been performed such as drug administration as previously explained that the decrease in blood pressure in the intervention group was higher than the control group who had the habit of taking medicine as many as 74%.

## Conclusion

In the present study, RESIK therapy significantly decreased blood pressure, dropping it from category 1 hypertension to pre-hypertension. RESIK therapy should be administered in accordance with the implementation protocol to obtain an optimal therapeutic effect.

## Data Availability

A confidentiality agreement with respondents prevent us from sharing the data.

## Abbreviations

ACE: Angiotensin-Converting Enzyme
ACTH: Adrenocorticotropic Hormone
BMI: Body Mass Index
CRH: Corticotrophin Releasing Hormone
MMSE: Mini-Mental State Exam
NCDs: Non-Communicable Diseases
NO: Nitric Oxide
NOF: New-Onset osteoporotic Fracture
RESIK: Progressive Muscle Relaxation and Music Therapy
SD: Standard Deviation
